# Remarkable Enhancement of Catalytic Reduction of Nitrophenol Isomers by Decoration of Ni Nanosheets with Cu Species

**DOI:** 10.1021/acsomega.4c04762

**Published:** 2024-08-27

**Authors:** Victoria Avalos-Ballester, Brenda Acosta, Elena Smolentseva

**Affiliations:** † Coordinación para la Innovación y la Aplicación de la Ciencia y la Tecnología, Universidad Autónoma de San Luis Potosí, Álvaro Obregón 64, San Luis Potosí, S.L.P. 78000, México; ‡ Investigadora por México CONAHCYT, Coordinación para la Innovación y la Aplicación de la Ciencia y la Tecnología, Universidad Autónoma de San Luis Potosí, Álvaro Obregón 64, San Luis Potosí, S.L.P. 78000, México; § Universidad Nacional Autónoma de México Centro de Nanociencias y Nanotecnología, Km. 107 Carretera Tijuana a Ensenada, C.P. 22860 Ensenada, B.C., México

## Abstract

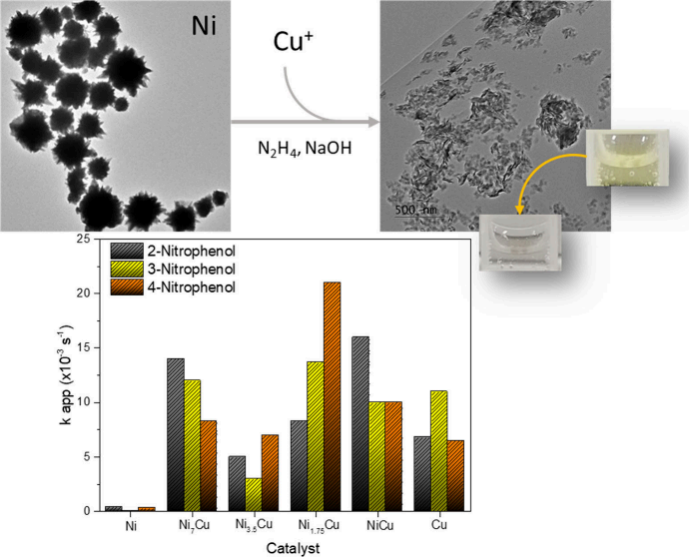

Herein, the catalytic reduction of isomers of nitrophenols (NPS) using Ni_*x*_Cu_*y*_ nanostructures with different molar ratios is presented. Ni_*x*_Cu_*y*_ catalysts are prepared using star-shaped Ni nanoparticles as seeds. The applied synthesis transforms Ni nanoparticles into sheet-like structures when Cu species are deposited on them. The bimetallic sheet-like Ni_*x*_Cu_*y*_ nanostructures demonstrate high catalytic activity to reduce NP isomers concerning their monometallic counterparts. The contribution of the Cu^+^ species affects the catalytic reduction of the NPS isomers. For example, the catalytic reduction of 4-nitrophenol (4-NP) depends on the Ni:Cu molar ratio: Ni_1.75_Cu > Cu > NiCu > Ni_7_Cu > Ni_3.5_Cu > Ni. The Ni_7_Cu catalyst exhibits the highest catalytic activity in the reduction of nitrophenol isomers 2-nitrophenol (2-NP) and 3-nitrophenol (3-NP), and the obtained results are comparable with those reported for noble-metal-based catalysts. The low-cost production of Ni_*x*_Cu_*y*_ catalysts and their high catalytic stability and availability make them attractive for industrial applications.

## 1 Introduction

The industrial use of nitrophenol compounds is important due to their wide range of applications, such as dyes, rubbers, fungicides, pesticides, explosives, and medicine manufacturing.^1^ On the other hand, nitrophenols (NPS) are strong contaminants whose degradation in soil and groundwater takes a long time.^2^ Additionally, exposure to these compounds causes health damage (stomach upset, weakness, confusion, rapid heartbeat, fever, etc.) and even death since they are recognized as highly toxic.^3−5^ Thus, NPS disposal is strongly required to prevent contact of NPS with the environment. Various approaches are applied to process nitrophenolic compounds, including absorption methods, extraction with solvents, advanced oxidative processes, use of microorganisms, and catalytic processes.^6^ However, bioremediation with microorganisms and oxidative processes is ineffective because these methods only transfer the NPS from one phase to another without eliminating them.^6^ Additionally, special reaction conditions, such as high temperature and pressure, are required, which raises the cost.

Thus, the catalytic transformation of NPS, when valuable intermediates may be produced,^6^ is an excellent alternative to other methods. The aminophenols (APS), products of the catalytic transformation of NPS, are categorized by high added value and less toxicity. Many APS are essential intermediates in the pharmaceutical, photographic, and plastic industries.^5^ Commonly, the catalytic transformation of NPS into APS proceeds via their reduction with hydrogen at room temperature. According to the reaction mechanism, metallic species are required to promote molecular hydrogen dissociation into atomic hydrogen.^7^ Noble-metal-based catalysts such as Au,^8,9^ Ag,^7,9,10^ Pd,^11−13^ and Pt^3,14^ are commonly used in the catalytic reduction of nitrophenols due to their high efficiency in hydrogen activation. However, the noble metals’ costliness, low catalytic stability, and low availability do not make them attractive for industrial applications.^3,12,13^ For this reason, non-noble metal catalysts have become widely spread during the last five years.^15^

Transition metals have been used in catalytic applications for many years. Thus, copper (Cu)-based catalysts are the most common for homocoupling reactions, the Ullmann reaction, the two-electron oxidation/reduction and arylation of arylamides, acrylamides, amides, and others.^16^ At the same time, nickel (Ni)-based catalysts promote the reduction of the nitrile group and the formation of new carbon–carbon bonds.^17^ However, for reactions in which hydrogen needs to be activated, the catalytic properties of Ni and Cu are not comparable with those found for noble metals. Therefore, a suitable alternative may be to combine Ni and Cu metals to improve their catalytic properties. Bimetallic catalysts based on transition metals are known to be characterized by a synergistic effect that magnifies their individual catalytic properties.^18^ For example, bimetallic catalysts such as NiMo and CoMo have high efficiency in the hydrodesulfurization of fuels.^19,20^

Ni is one of the most abundant transition metals on the planet, with electronic properties comparable to those of the noble Pd and Pt metals.^21^ Indeed, Ni nanoparticles received considerable attention as catalysts for several reactions such as nitrobenzene and NPS hydrogenation,^22−25^ oxygen reduction,^26^ olefin oxidation,^27^ and ketone reduction^28^ due to their high catalytic efficiency. Ni nanocrystals with different crystalline structures, such as face-centered cubic (FCC) and hexagonal close-packed (HCP), have been evaluated in the catalytic reduction of NPS isomers with temperature variations (25, 35, 45, and 55 °C). The experiments revealed that Ni nanocrystals could catalyze the hydrogenation of NPS efficiently. The best results were obtained using Ni nanocrystals with an HCP structure at 55 °C for NPS isomers (2-NP, 3-NP, and 4-NP).^29^

Moreover, Ni easily forms alloys with both noble metals (Au, Ag, Pt, Pd, Ru, among others) and many transition metals (Co, Fe, Cu, Cr). The effect of the mass and the molar ratios between Ni and other metallic elements was used to develop multiple Ni-based systems for various catalytic applications.^21^ Industrial applications of Ni-based bimetals include bioimaging, sensing, drug delivery, biomedicine and therapeutic applications (magneto-plasmonic alloys Ni–Au, Ni–Ag),^30^ coatings for corrosion resistance, conductive paints (Ni–Cu, Ni–Fe, Ni–Co),^31−34^ and fuel electrodes and electrochemical biosensors (Ni–Pt).^35,36^ Ni–M (M = Mn, Fe, Co, Cu, Ru, Rh, Pd, Ag, Ir, Pt, Au) bimetallic nanoparticles have many catalytic approaches such as hydrolysis of ammonia borane, hydrodechlorination, CO oxidation, electrocatalysis, hydrogenation of sugar derivatives, water gas shift, reforming of oxygenates or hydrocarbons, etc.^21^ In addition, the impact of the Ni morphology on its catalytic properties was reported using various structure modifiers.^22^ The obtained results demonstrated that the formation of the Cu–Ni alloy permits diminished agglomeration of the particles. It is reported that Cu affects Ni dispersion and can even retard the poisoning of Ni.^37,38^

Ni–Cu bimetallic (equimolar ratio) spherical nanoparticles supported on ginger powder were studied in the reduction of NPS and organic dyes, being more effective in 4-NP reduction.^39^ Additionally, a dramatic enhancement in the catalytic reduction of 4-NP on Cu@Ni bimetallic nanowires supported on graphene was obtained using a Ni:Cu molar ratio of 5:1. The results were attributed to the synergetic effect between the metals and the electron transfer from the graphene to the metals.^40^ Free CuNi nanocrystals with different molar ratios were studied in the catalytic reduction of 4-NP. The synergistic effect of Ni and Cu was confirmed, manifesting higher activity of the bimetallic catalyst with the optimal ratio of Cu_3_Ni_2_ in contrast with their monometallic counterparts.^41^

In this regard, since toxic structural NPS isomers exhibit different atomic arrangements (with charge delocalization affecting both the intra- and intermolecular interactions), it is very interesting to discuss the intrinsic effect of Ni–Cu-based catalysts where the metal molar ratios may be fine-tuned. Moreover, the analysis of unsupported bimetallic anisotropic morphologies for the catalytic reduction of NPS isomers remains a challenge. Therefore, having an understanding of the combined effect of both the fine-tuned metallic molar ratio and the morphology of Ni–Cu-based catalysts is crucial for the reduction of NPS isomers.

This work presents the synthesis of Ni nanostars for their further partial decoration with Cu (Ni_*x*_Cu_*y*_) via a low-cost colloidal deposition process. A sheet-like Ni_*x*_Cu_*y*_ morphology, derived from the collapse of the Ni seeds under the Cu deposition conditions, is described. The high dispersion of the metallic species promotes a synergistic effect between Ni and Cu, enhancing the Ni_*x*_Cu_*y*_ catalytic properties. The effect of the Ni:Cu molar ratio achieved for each case was analyzed in the catalytic reduction of the group of NPS isomers, 2-, 3-, and 4-nitrophenol, into their corresponding amino derivatives (2-, 3-, and 4-aminophenol, respectively). The results revealed the presence of cationic and metallic species whose transformation under reaction conditions correlates with the catalytic performance of the bimetallic Ni_*x*_Cu_*y*_ nanostructures. These findings help us understand the role of oxidized Ni and Cu species in the catalytic reduction of 4-NP. Compared to similar catalysts reported in the literature, the Ni_*x*_Cu_*y*_ catalysts demonstrated remarkable improvement in catalytic activity, surpassing even noble-metal-based catalysts. The low-cost production, high catalytic stability, and availability of Ni_*x*_Cu_*y*_ catalysts make them attractive for industrial applications, especially when compared to noble-metal-based catalysts.

## 2 Experimental Section

### 2.1 Reagents

Nickel nitrate hexahydrate (Ni(NO_3_)_2_·6H_2_O, 97%), copper nitrate hemipentahydrate (Cu(NO_3_)_2_·2.5H_2_O, 98%), hydrazine monohydrate (N_2_H_4_·H_2_O, 98%), cetyltrimethylammonium bromide (C_19_H_42_BrN, 98%), sodium hydroxide (NaOH, 98%), ethylene glycol (C_2_H_6_O_2_, 99%), sodium borohydride (NaBH_4_, 99%), 2-nitrophenol (C_6_H_5_NO_3_, 99%), 3-nitrophenol (C_6_H_5_NO_3_, 99%), and 4-nitrophenol (C_6_H_5_NO_3_, 99%) were purchased from Sigma-Aldrich. Absolute ethanol (C_2_H_6_O, 99.5%) and deionized water (H_2_O-DI, analytical reagent) were provided by Golden Bell and MAPLA, respectively. All of the reagents were used as received.

### 2.2 Catalyst Preparation

#### 2.2.1 Synthesis of Nickel Nanoparticles

The synthesis reported here adopted the methodology described by Lin et al.^42^ with some modifications. Ni nanoparticles were prepared via the reduction of Ni(NO_3_)_2_·6H_2_O previously dissolved in C_2_H_6_O_2_ (47 mM, 20 mL) with N_2_H_4_·H_2_O (2.7 M, 9 mL). After the color changed from green to purple, NaOH (1 M, 5 mL) aqueous solution was added drop by drop. The reaction proceeded for 1 h under magnetic stirring at room temperature. Immediately afterward, the temperature was raised to 60 °C and kept for 2 h. The metallic Ni nanoparticle formation was accompanied by gradual changes in the suspension color until it became black. The obtained suspension was cooled. The formed Ni nanoparticles were collected by centrifugation. Then, the Ni nanoparticles were rinsed twice with C_2_H_6_O and deionized water. The wet Ni nanoparticles were redispersed in a C_19_H_42_BrN solution (0.1 M, 40 mL) and magnetically mixed for 12 h. Finally, the Ni nanoparticles were recovered by centrifugation, rinsed with C_2_H_6_O and deionized water, and dried overnight at 80 °C. Obtained powdered samples of Ni nanoparticles were labeled as Ni.

#### 2.2.2 Synthesis of Ni_*x*_Cu_*y*_ Nanoparticles

Ni_*x*_Cu_*y*_ bimetallic nanoparticles were synthesized using a successive two-step method, similar that used by Lin et al.^42^ First, previously obtained Ni nanoparticles (1 × 10^–3^ mol) were dispersed in C_2_H_6_O (20 mL). Then, a specific amount of Cu(NO_3_)_2_·2.5H_2_O was added to obtain Ni_*x*_Cu_*y*_ samples with different molar ratios between Ni and Cu metals. The formed suspension was magnetically stirred for 1 h, and then NaOH solution (1 M, 5 mL) was added dropwise. Immediately afterward, the temperature was raised to 80 °C and kept for 2 h. In this step, the solution color changed from black to metallic gray, indicating CuO formation.^42^ The obtained suspension was cooled, and the Ni–CuO nanoparticles were collected by centrifugation. The Ni–CuO nanoparticles were rinsed twice with C_2_H_6_O and deionized water. Then, the wet Ni–CuO sample was redispersed in C_19_H_42_BrN (0.1 M, 40 mL) and N_2_H_4_·H_2_O (1.5 M, 6 mL) solution. The obtained suspension was mixed for 12 h at room temperature. Again, the color of the suspension changed from metallic silver to pink, confirming metallic Cu formation.^42^ Ni_*x*_Cu_*y*_ bimetallic nanoparticles were collected by centrifugation and rinsed twice with C_2_H_6_O and deionized water. Obtained samples were dried overnight at 80 °C. The powder catalysts were labeled as Ni_7_Cu, Ni_3.5_Cu, Ni_1.75_Cu, and NiCu (1:1 molar ratio), considering the Ni and Cu molar ratio confirmed by inductively coupled plasma–optical emission spectroscopy (ICP-OES) analysis.

#### 2.2.3 Synthesis of Cu Nanoparticles

The preparation of these reference materials was also carried out on the basis of the method described by Lin et al.^42^ In brief, Cu(NO_3_)_2_·2.5H_2_O was dissolved in C_2_H_6_O (47 mM, 20 mL) and mixed with N_2_H_4_·H_2_O (0.9M, 6 mL). Subsequently, a NaOH solution was added dropwise (1 M, 5 mL). Immediately afterward, the temperature was raised to 80 °C and kept for 2 h. After this time, the color solution changed from blue (typical for Cu(NO_3_)_2_·2.5H_2_O solution) to pink, indicating metallic Cu formation. Cu nanoparticles were collected by centrifugation and rinsed twice with C_2_H_6_O and deionized water. Then, the wet Cu nanoparticles were redispersed in a C_19_H_42_BrN solution (0.1 M, 40 mL). The formed suspension was mixed for 12 h at room temperature. Finally, the Cu sample was collected by centrifugation, rinsed with C_2_H_6_O and deionized water, and dried overnight at 80 °C. The powder Cu catalyst was labeled as Cu.

### 2.3 Catalyst Characterization

The chemical composition of the catalysts was analyzed by ICP-OES using Vista-MPX CCD simultaneous equipment (Varian). A calibration curve was prepared with Ni and Cu standards. Typically, 6 mg of solid sample was treated in an acid mixture HNO_3_–HCl–HF in a ratio of 1:1:1. After that, the sample was diluted with water and analyzed. Measurements were carried out in triplicate, and blanks were handled using the same procedure. The morphology of the Ni, Cu, and Ni_*x*_Cu_*y*_ nanoparticles was characterized by transmission electron microscopy (TEM) using a JEM-2100 instrument (JEOL) and high-resolution (HR)-TEM in a FEI TECNAI F30 instrument operating at 200 and 300 kV, respectively, coupled with an EDX detector. Prior to the analysis, the sample was suspended in isopropyl alcohol and then a couple of drops were added to a copper grid covered by a lacey/carbon (200 mesh). The crystalline structure of the samples was characterized by X-ray diffraction (XRD) using an Empyrean-Malvern diffractometer (Panalytical) with Kα = 1.54 Å radiation from a copper X-ray tube, operating at 30 mA and 40 kV. The Raman spectra were recorded using an Xplora One micro-Raman spectrometer (HORIBA), equipped with a Syncerity detector and a green laser (532 nm), at 20 mW of power. The Raman spectrometer was coupled to an optical microscope, an Olympus BX41. The signal was recorded by a cooled CCD detector at 70 °C. The laser beam was focused on the sample using an Olympus 20LWD objective to give a slit of 100 μm and a hole of 300 μm on the sample. The data acquisition time was 20 s/scan, collecting four co-added scans at a monochromator grating of 1200 grooves mm^–1^. The electronic states for each metal were studied by X-ray photoelectron spectroscopy (XPS) using a SPECS spectrometer equipped with a PHOIBOS 150 WAL hemispherical analyzer and an Al Kα (1486.6 eV) monochromatic source. The optical properties of Ni_*x*_Cu_*y*_ catalysts were studied by UV–vis spectroscopy using a UV-3600 UV–vis–NIR Plus spectrophotometer (Shimadzu) in a wavelength range of 200–900 nm, with a 2 mm path length quartz cell as a sampler holder.

### 2.4 Catalytic Activity Measurement

The promotional effect of Ni nanostructures decorated with Cu was evaluated in the reduction of the 4-, 3-, and 2-NPS isomers (4-NP, 3-NP, and 2-NP) into 4-, 3-, and 2-APS (4-AP, 3-AP, and 2-AP), respectively, in excess NaBH_4_ at 25 °C. Catalytic reactions were monitored via *in situ* UV–vis spectroscopy using an ultrafast Ava-Spec-ULS40952 UV–vis spectrophotometer (Avantes) equipped with an AvaLight-DH-S light source and a CUV-UV/vis-TC chamber that permits temperature and agitation control.

The protocol used for the catalytic reduction of the NPS isomers was previously described.^43,44^ In a typical catalytic run, the aqueous solution of the NPS isomer (10 μL, 30 mM) and NaBH_4_ (3.7 mL, 0.1 M) was stirred in the quartz cuvette cell (1 cm in path length) for 15 min. Then, 20 μL of the catalyst aqueous suspension (1.4 mg, 1 mL) was added to the cuvette. The reaction proceeded at 25 °C under magnetic stirring (1200 rpm). Each spectrum was recorded automatically every 2 s.

## 3 Results and Discussion

### 3.1 Catalyst Characterization

**Figure 1:**
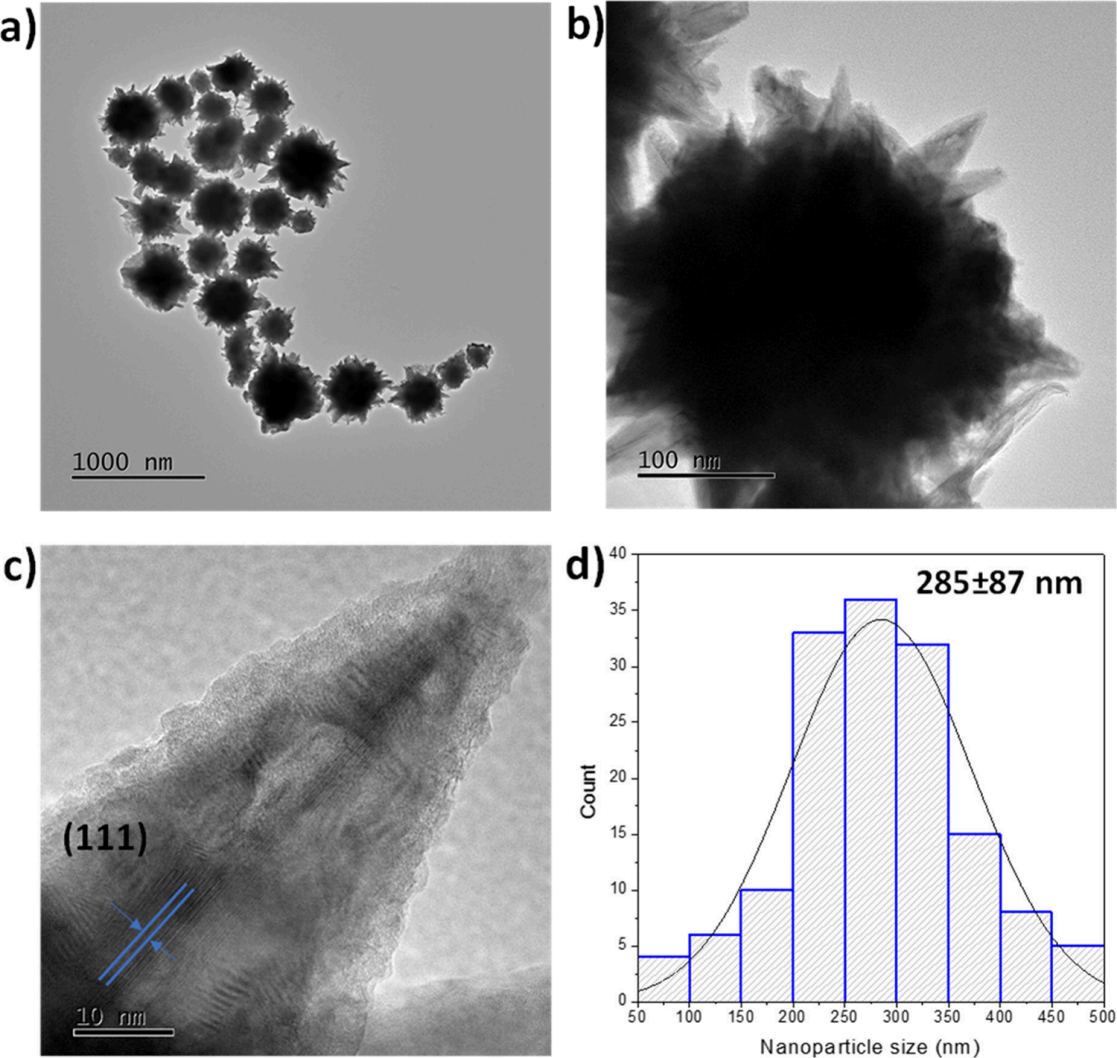
Typical TEM micrographs of Ni nanostructures at (a) low and (b) high magnification and (c) HR-TEM image. (d) Size distribution histogram of star-shaped Ni nanostructures.

Figure 1 shows typical TEM images of the as-obtained Ni nanoparticles and their corresponding nanoparticle size distribution histogram. The applied synthesis conditions resulted in star-shaped Ni nanoparticles as the predominant morphology (Figure 1a–c). As shown in Figure 1c, the interplanar distances revealed the (111) plane indexation characteristic for metallic Ni with an FCC structure.^45^ Furthermore, the assembled Ni nanostructures demonstrated a broad size distribution from 60 to 600 nm (Figure 1d) with an average diameter of 285 nm. According to the literature, similar synthesis conditions result in spherical Ni nanoparticles.^46^ Meanwhile, the star-shaped Ni morphology is found under relatively harsh synthesis conditions (15 M NaOH and ethylenediamine as solvent)^47^ or microwave irradiation.^48^

It is known that the Ni nanostructures are formed via the aggregation of Ni seeds (∼3–4 nm) stimulated by the intrinsic magnetism of metallic Ni.^49^ In this case, the obtained star-shaped Ni nanoparticles may be associated with the use of NaOH as a hydrazine activator and CTAB as a stabilizing agent. Indeed, the presence of NaOH during the Ni nanoparticle synthesis may promote partial Ni hydrolysis, leading to the formation of smaller Ni nanostructures, such as sheets.^50^ Thus, it is possible to propose that the recently formed Ni nanoparticles were partially hydrolyzed, resulting in sheet-like structures. These Ni nanosheets were then stacked on the larger Ni spherical nanoparticles, directed by the presence of CTAB that had previously adsorbed on the Ni surface. It is well-known that CTAB acts as a structure modifier due to its deposition on specific superficial sites, promoting the formation of particular morphologies.^51,52^

Figure S1 shows a typical micrograph of the prepared Cu nanoparticles. It was found that Cu nanoparticles were formed from the aggregation of smaller Cu species with a size of ca. 10 nm. According to ref (53), Cu nanoparticles of around 20 nm tend to form agglomerates of defined sizes and shapes, directed by the structure tracer used. Indeed, the synthesized Cu nanoparticles resulted in a quasi-spherical morphology with a size of ∼200 nm (see Figure S1).

**Figure 2:**
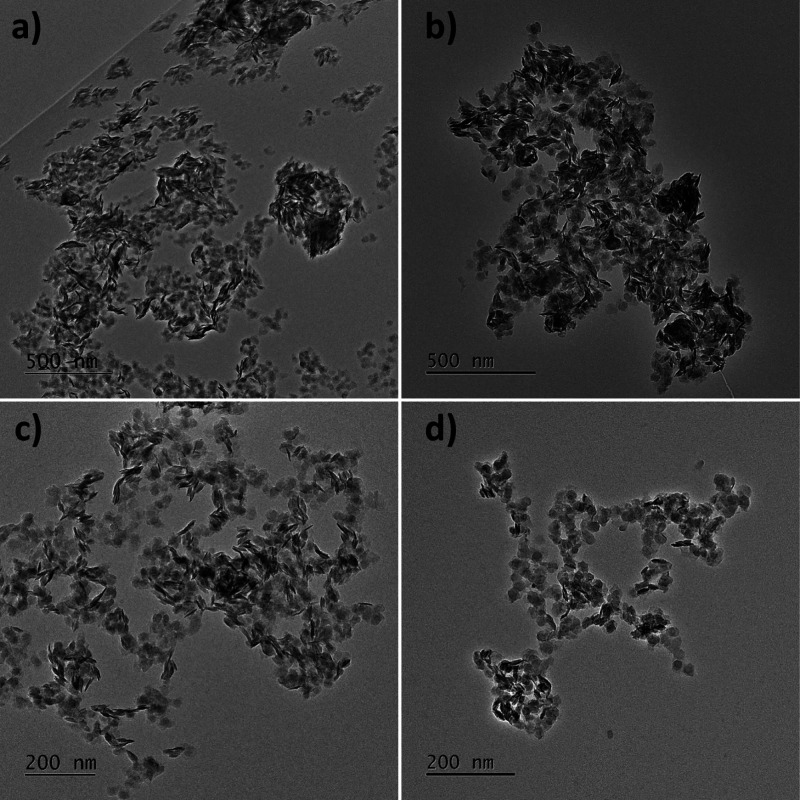
TEM images of Ni_*x*_Cu_*y*_ samples with different molar ratios: (a) Ni_7_Cu, (b) Ni_3.5_Cu, (c) Ni_1.75_Cu, and (d) NiCu (1:1 molar ratio).

Figure 2 presents typical TEM images of the bimetallic Ni_*x*_Cu_*y*_ nanostructures. The deposition of Cu on the Ni nanostructures, used as seeds, led to the dramatic transformation of the initial star-shaped Ni morphology, independent of the amount of Cu deposited in each case. Similar results were reported for Ni_2_Cu_3_ nanoparticles with a well-defined quasi-spherical morphology.^41^ The synthesis of bimetallic catalysts can be divided into two steps: (1) seed growth and (2) one-pot co-reduction of precursor salts. In the first case, a core–shell-type structure is obtained because the reduction of the second metal proceeds in the presence of the first metal seeds. In the second case, two metallic precursors are mixed and then reduced with the possible formation of alloys due to the simultaneous reduction of metallic precursors.^21^ In the present work, the formation of a core–shell configuration was expected in accordance with the methodology used. However, according to the results presented in Figure 2, a sheet-like morphology was obtained instead of stars. As mentioned earlier, the use of NaOH promotes the dissolution of Ni nanostructures. Additionally, the presence of the Cu precursor interferes with the Ni magnetism, preventing the rearrangement of Ni.^41^ Note that the TEM image in *Z* contrast (Figure S2) shows both brilliant and blurry zones. The bright sections in *Z* contrast are commonly attributed to metallic species.^54^ Thus, the analyzed sample involves both metallic and nonmetallic components, suggesting that oxidized Cu may be formed. The EDX elemental mapping analysis (Figure S2) confirmed the presence of both metals in the bimetallic samples.

**Figure 3:**
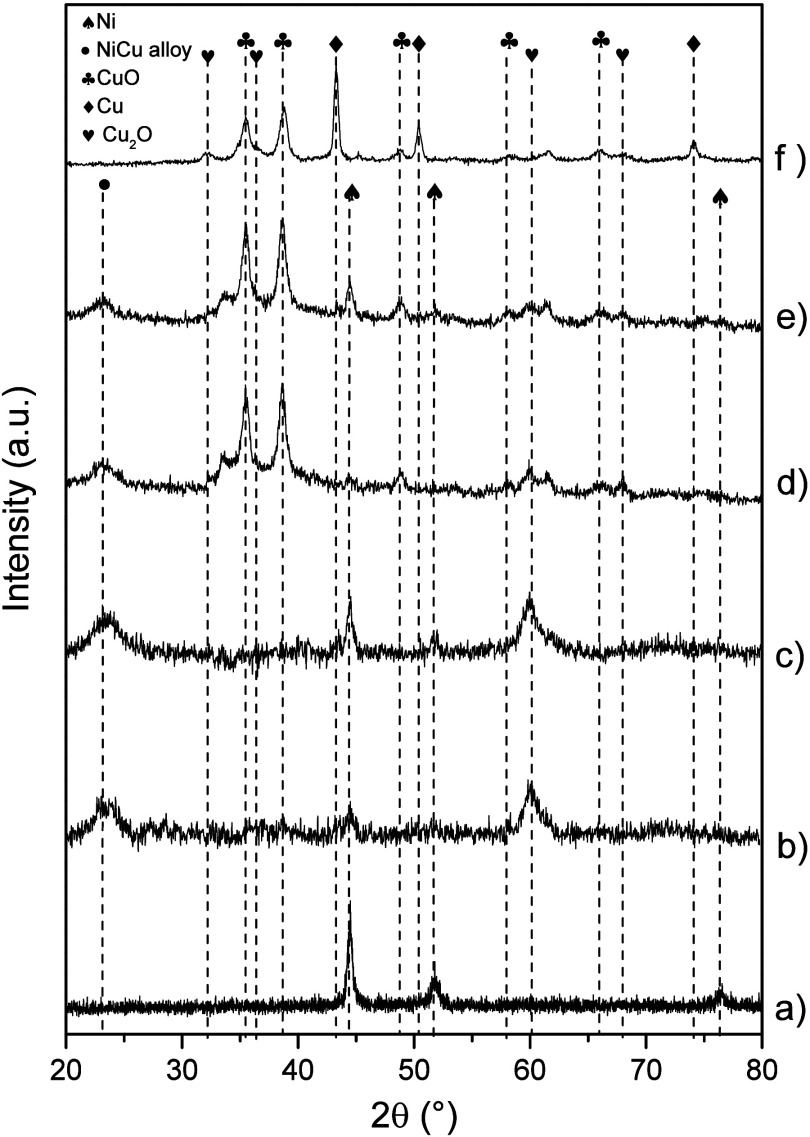
XRD patterns of Ni, Cu, and Ni_*x*_Cu_*y*_ samples: (a) Ni, (b) Ni_7_Cu, (c) Ni_3.5_Cu, (d) Ni_1.75_Cu, (e) NiCu, and (f) Cu.

Figure 3 summarizes the XRD patterns for the mono- and bimetallic samples. Well-defined peaks centered at 45° and 52.3°, along with other broad signals at 77.4° 2θ, were observed in the diffractogram for the star-shaped Ni nanostructures (Figure 3a). These peaks correspond to the (111), (200), and (222) crystallographic planes, respectively, for metallic Ni with an FCC structure according to the JCPDS 03-1051 chart. As expected, the synthesis of monometallic Ni nanoparticles resulted in the complete reduction of the Ni precursor into metallic Ni.

On the other hand, the XRD pattern for Cu nanoparticles (Figure 3f) revealed peaks corresponding to three different crystalline phases: metallic Cu, Cu_2_O, and CuO. Peaks at 2θ equal to 43.3°, 50.3°, and 74.15° were assigned to metallic Cu with an FCC structure, associated with the Miller indexes (111), (200), and (220), respectively, according to the crystallographic JCPDS 85-1326 chart. Some peaks related to Cu_2_O were also found at 32.1°, 36.3°, 61.5°, and 68.2° 2θ, correlated with the (110), (111), (220), and (311) crystallographic planes of the primitive cubic phase of Cu_2_O (JCPDS card 75-1531). Finally, the peaks at 35.4°, 38.8°, 48.9°, 58.23°, and 66° 2θ corresponded to the planes (002), (200), (2̅02), (202), and (022), respectively, and were attributed to the crystalline monoclinic structure of CuO nanoparticles (JCPDS 80-1916). The synthesis of metallic Cu nanoparticles by chemical reduction commonly results in different crystalline phases mainly associated with Cu oxides.^55^ This is due to the tendency of Cu to be oxidized when in contact with the environment and the precursors used for the synthesis.^56,57^

The XRD patterns for bimetallic Ni_*x*_Cu_*y*_ samples presented a new broad peak at 23° 2θ, associated with copper–nickel alloys (Figure 3b–e).^58^ Note that the intensity of the peaks corresponding to CuO and Cu_2_O in bimetallic Ni_*x*_Cu_*y*_ nanostructures increased with increasing amounts of Cu in the samples. No peaks attributed to metallic Cu were detected for the bimetallic Ni_*x*_Cu_*y*_ samples. Thus, the samples demonstrated a homogeneous dispersion of Ni and Cu as nanosheets, which could be accompanied by some CuO or Cu_2_O nanoparticles. Additionally, the XRD patterns of bimetallic Ni_*x*_Cu_*y*_ nanostructures revealed their poor crystallinity compared to the monometallic Ni or Cu nanoparticles. Crystallite sizes for bimetallic nanostructures were estimated for the peak at 45° 2θ, corresponding to the (111) plane of Ni FCC (Table S1). The star-like Ni nanoparticles presented a crystallite size of 18.7 nm. However, the addition of Cu led to lower crystallite sizes. It seems that the changes in the morphology from star-like to sheets affected the crystal stacking, decreasing the crystallite size. Thus, the effective decoration of Ni metallic nanostructures with Cu species at different molar ratios was confirmed. It is proposed that the hydrolysis of Ni under Cu deposition conditions caused the changes in morphology, crystallinity, and interaction between metals. So the Ni_*x*_Cu_*y*_ nanosheets prepared in this work contained metallic Ni, CuO, Cu_2_O species, and Ni–Cu alloys.

**Figure 4:**
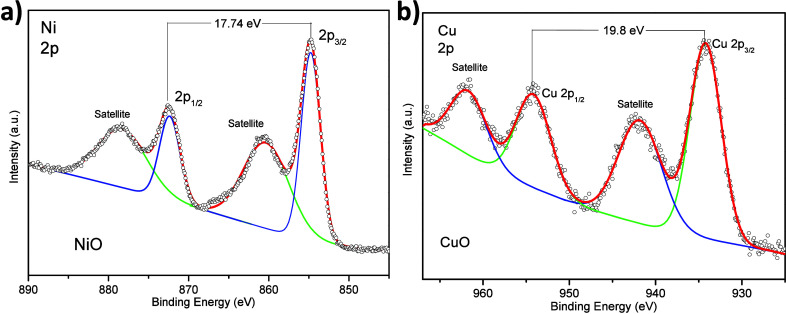
XPS spectra of the Ni_1.75_Cu catalyst: (a) Cu 2p and (b) Ni 2p. The red lines denote the fitting line for the XPS spectra, and the green and blue lines represent the deconvoluted spectra for the respective elements and satellites.

Figure 4 presents the XPS spectra for the Ni_1.75_Cu sample. The survey spectrum (Figure S3) confirmed the presence of Ni, Cu, and O in the catalyst. The Ni 2p high-resolution spectrum (Figure 4a), separated by 17.74 eV, manifested two spin–orbit components corresponding to Ni 2p_1/2_ and Ni 2p_3/2_ centered at 873 and 855 eV, respectively, attributed to the Ni^2+^ chemical state.^59,60^ These peaks were accompanied by two satellites at 878.7 and 860.7 eV. XPS data revealed the presence of NiO particles. However, the XRD data evidenced only metallic nickel. It is possible that NiO was formed as a thin layer on the Ni_*x*_Cu_*y*_ nanostructures.

Finally, Figure 4b displays the high-resolution spectrum corresponding to Cu 2p. The spectrum showed two peaks assigned to Cu 2p_3/2_ and Cu 2p_1/2_ centered at 934.4 and 954.4 eV, respectively, attributed to the Cu^2+^ chemical state.^60^ These peaks were accompanied by two satellite peaks at 942 and 962 eV. The detailed analysis of the survey spectrum revealed the presence of a signal at 570 eV (Cu LMM) that corresponds to Cu_2_O in the catalyst (Figure S3). Thus, it may be concluded that the main Cu species were cationic Cu^δ+^ rather than metallic ones. The identical signals related to Ni and Cu were obtained for other Ni_*x*_Cu_*y*_ catalysts with different molar ratios (see Figure S4).

**Figure 5:**
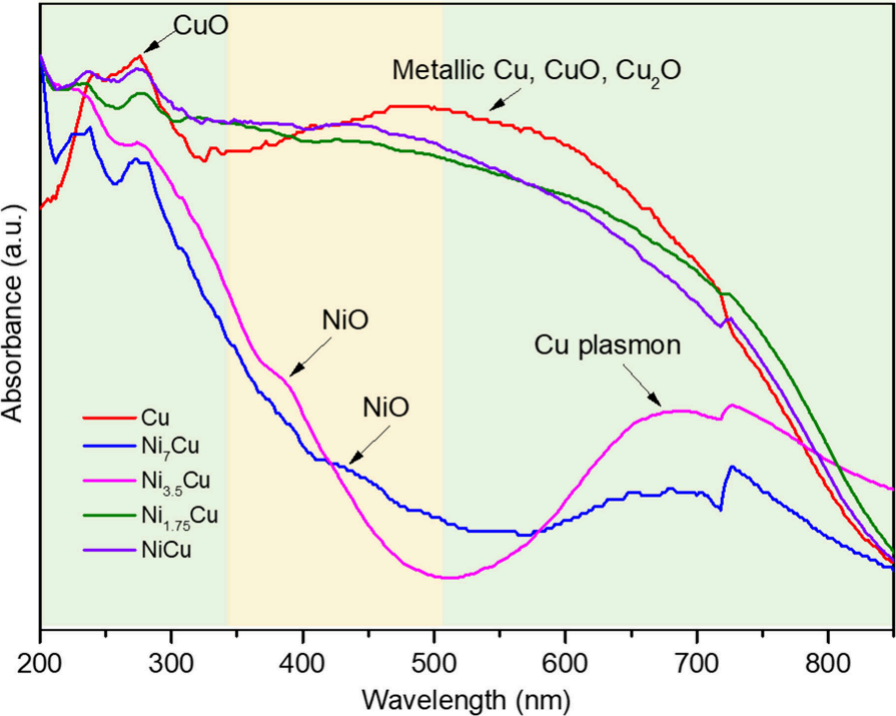
UV–vis absorption spectra of the Cu and Ni_*x*_Cu_*y*_ samples.

Figure 5 illustrates the UV–vis absorption spectra of Cu nanoparticles and Ni_*x*_Cu_*y*_ catalysts. Absorbance bands associated with cationic Ni^2+^ (band enclosed in the yellow box in Figure 5) and metallic Cu and cationic Cu^2+^ and Cu^+^ species (bands enclosed in the green boxes in Figure 5) were found. All studied samples presented an absorption band around 275 nm, commonly assigned to CuO.^61^ Note that the absorption spectra of Ni_*x*_Cu_*y*_ catalysts with the lowest Cu content (Ni_3.5_Cu and Ni_7_Cu) demonstrated a well-defined band between 600 and 800 nm, attributed to the electron d–d transitions in Cu^2+^ in a distorted octahedral surrounded by oxygen in CuO particles.^62^ Meanwhile, the samples with the highest Cu content presented a broad adsorption band in the range 350–800 nm, partially overlapping with the band assigned to Ni^2+^. Some reports attributed this band to the contribution of metallic copper species^63^ and cationic ones for CuO and Cu_2_O, respectively.^64^

For Ni_1.75_Cu and NiCu samples, a blue band shift at 350–800 nm for metallic Cu species was observed in the UV–vis spectra. This was attributed to the increase of the Cu^2+^ species presented in the samples.^64^ Additionally, some Ni species could contribute to this shift, which was found for the Ni_7_Cu and Ni_3.5_Cu samples. The transition from 2p orbitals of O^2–^ to the 3d orbitals in Ni^2+^ and the internal d–d transition in the Ni host lattice resulted in shoulders in the 400–580 nm range.^65^ UV–vis spectra for Ni_7_Cu and Ni_3.5_Cu presented shoulders with different intensities at 365 and 425 nm (see Figure 5), commonly assigned to Ni in a tetrahedral coordination.^66^ On the other hand, it is reported that the intensity of the band at 365 nm, related to NiO, increases when the particle size of those species decreases.^67^ Thus, the prepared catalysts demonstrated different Cu and Ni species, and their contribution depended on the sample composition. The samples with low Cu content resulted in the formation of metallic Cu species only, while an increase in the Ni concentration in the samples led to the formation of NiO particles.

**Figure 6:**
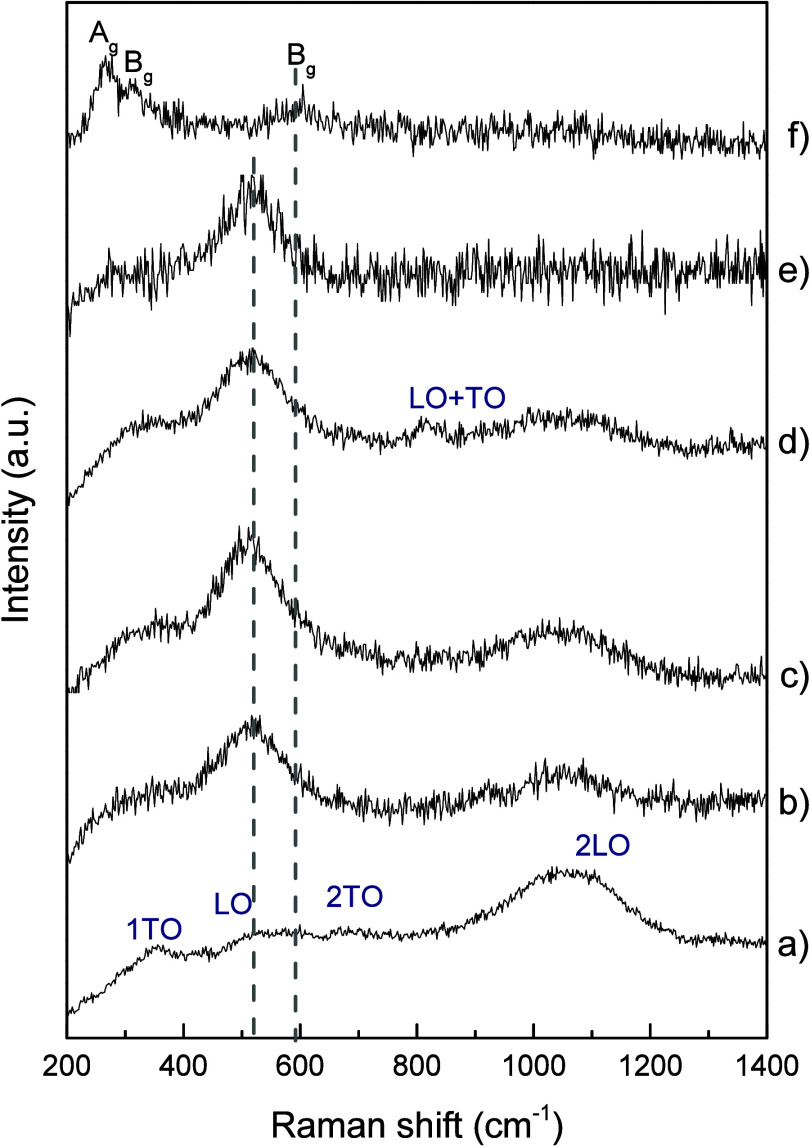
Raman spectra of Ni, Cu, and Ni_*x*_Cu_*y*_ samples: (a) Ni, (b) Ni_7_Cu, (c) Ni_3.5_Cu, (d) Ni_1.75_Cu, (e) NiCu, and (f) Cu.

Figure 6 shows the Raman spectra obtained for the mono- and bimetallic Ni_*x*_Cu_*y*_ catalysts. The Raman spectrum corresponding to the Ni nanoparticles exhibited typical NiO bands. The band at around 515 cm^–1^ belonged to the first-order longitudinal-optical (LO) mode of a phonon, associated with Ni–O vibrations; meanwhile, the broader band at ∼1060 cm^–1^ corresponded to the second-order longitudinal-optical two-phonon (2LO) mode.^68^ The peaks related to transverse-optical (TO) modes were located at ∼367 cm^–1^ and ∼704 cm^–1^ for the first-order (1TO) and second-order (2TO) phonon modes, respectively. The band at ∼896 cm^–1^ was assigned to the stretching modes of NiO (LO + TO).^68^

On the other hand, the Raman spectrum corresponding to the Cu nanoparticles exhibited three bands at around 265, 317, and 600 cm^–1^, typical of the monoclinic phase of CuO,^69^ which was in agreement with the results obtained by X-ray diffraction. The Raman spectrum of Cu nanoparticles (Figure 6f) presented the Ag^+^ 2B_g_ Raman active modes characteristic for CuO. The Ag^+^ mode corresponds to phase rotations, while the first B_g_ mode is due to the bending of CuO and the second B_g_ mode corresponds to the symmetrical stretching of oxygen.^69^ The Raman spectra of the bimetallic Ni_*x*_Cu_*y*_ catalysts presented the expected response of NiO–CuO mixtures.^37,70^ Specifically, the band between 400 and 650 cm^–1^ represented a contribution from the B_g_ bands of CuO and LO bands of NiO, indicating the formation of bimetallic Ni_*x*_Cu_*y*_ nanoparticles.^37,70^ This observation was consistent with the results obtained by XRD and XPS. It may be concluded that the bimetallic Ni_*x*_Cu_*y*_ catalysts contain a NiO–CuO mixture.

### 3.2 Catalytic Activity

The catalytic activity of the prepared nanostructures was evaluated in the reduction of NPS isomers used as model reactions. The NPS isomers are characterized by a well-detectable band in the UV–vis region that permits facile concentration analysis using UV–vis spectroscopy.^71−73^ 4-NP in contact with NaBH_4_ leads to the immediate formation of the 4-nitrophenolate ion (4-NPt), causing a bathochromic shift in the UV–vis spectrum from 316 to 400 nm.^72,73^ There are many kinetic models used to analyze the catalytic reduction of 4-NP. The models consider the role of the reaction parameters, such as the concentration of the reagents, catalyst mass, reaction temperature, adsorption model of reagents, and even the presence of intermediates.^74−76^ Basically, all models conclude that the kinetics of the reaction can be described in terms of the kinetic constant *k* related to the surface reactivity and the thermodynamics adsorption constants for both reagents (4NP, NaBH_4_). The use of excess NaBH_4_ provokes the reaction to be strongly conducted by the surface reactivity of the catalysts,^75^ making the reduction of 4-NP the slowest rate-determining step. Therefore, under this condition, the reaction is commonly analyzed by a pseudo-first-order kinetic model. The rate equation could be presented as
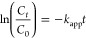
where *C*_*t*_ is the concentration of NPS at reaction time *t* and C_0_ corresponds to the initial concentration of NPS. Since the reaction medium obeys Beer–Lambert’s law, the absorbance of NPS UV–vis spectra represents their concentration. Hence, the rate equation can be rewritten as
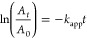
The apparent reaction rate constant (*k*_app_) may be estimated through the linear slope of the changes of the relative absorbance in logarithmic form versus the reaction time.^43,44^

**Figure 7:**
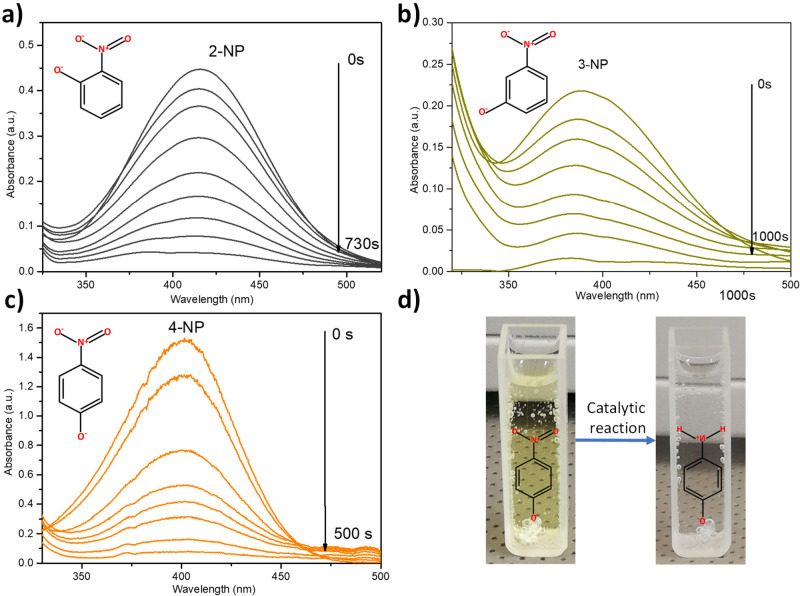
Successive UV–vis spectra of the reduction of NPS isomers in the presence of NaBH_4_ using Ni_1.75_Cu catalyst: (a) 2-NP, (b) 3-NP, and (c) 4-NP. (d) Photograph of the reaction cuvette before and after reduction of 4-NP.

Figure 7 summarizes the typical spectra collected *in situ* during the catalytic transformation of 2-NP, 3-NP, and 4-NP using Ni_*x*_Cu_*y*_ catalysts. The gradual decay of the notable peak of the NPS centered at 417, 393, and 400 nm for 2-NP, 3-NP, and 4-NP, respectively, was observed. The complete reduction of the NPS isomers was accompanied by the remarkable decolorization of the reaction solution (Figure 7d), which evidenced that the reaction ended.

**Figure 8:**
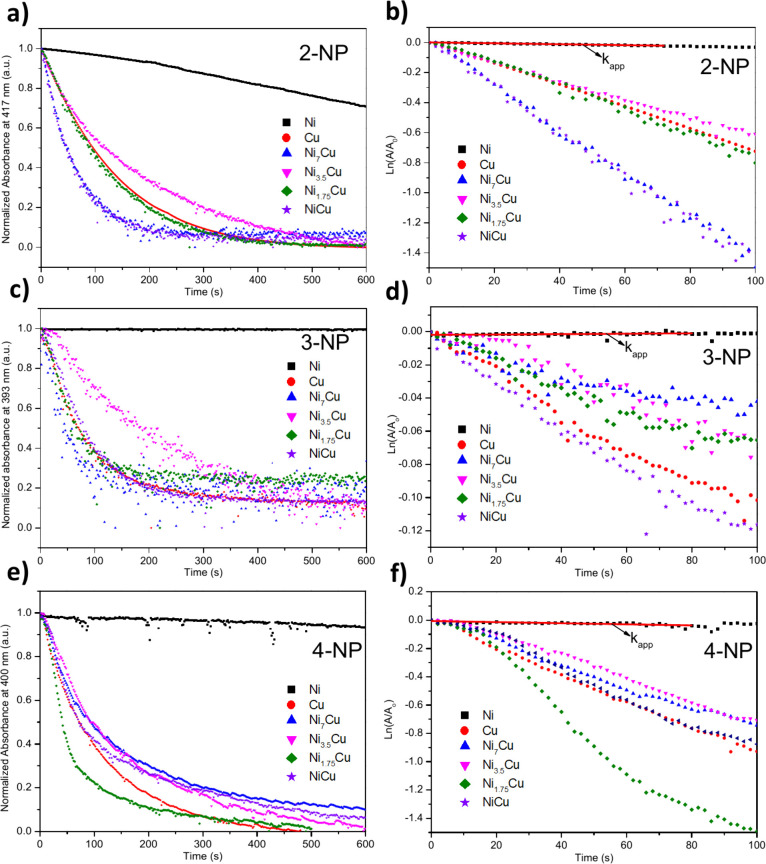
Kinetic analysis for the reduction of NPS isomers using Ni, Cu, and Ni_*x*_Cu_*y*_ catalysts. The changes in the relative absorbance versus reaction time for NPS reduction: (a) 2-NP, (c) 3-NP, and (e) 4-NP. Plot of ln(*A*/*A*_0_) versus reaction time for NPS reduction: (b) 2-NP, (d) 3-NP, and (f) 4-NP.

Figure 8 presents the kinetic analysis of NPS isomer reduction using Ni, Cu, and Ni_*x*_Cu_*y*_ catalysts, monitored by UV–vis spectroscopy *in situ*. The efficiency of the catalysts depended on the NPS isomer molecule structure and the composition of the catalysts used in each case. The estimation of *k*_app_ (Table 1) revealed the poorest catalytic activity of Ni nanostars in the reduction of all NPS isomers. This may be attributed to the large size of Ni nanostars (∼285 nm) and their intrinsic magnetism.^49^ Indeed, at the end of the reaction, the Ni sample was stuck to the magnet used for stirring the reaction medium. In contrast, monometallic Cu and bimetallic Ni_*x*_Cu_*y*_ nanostructures were well-dispersed in the reaction medium during the reaction.

**Table 1:** Comparison of Catalytic Activity in the Reduction of NPS Isomers Catalyzed by the Presently Prepared Catalysts and Those Reported in the Literature

isomer molecule	catalyst	apparent reaction rate constant *k*_app_ (×10^–3^ s^–1^)	activity parameter *K* (×10^–3^ s^–1^ μmol^–1^)	ref
4-nitrophenol	Ag/γ-Al_2_O_3_ calcinated	8.3	89.24	(71)
	Ni/γ-Al_2_O_3_ calcinated	2.3	13.52	(71)
	CoMoO_4_	4.8	0.01	(78)
	Ni/C black	10.0	0.53	(79)
	FCC Ni NCs^a^	2.2	0.65	(29)
	HCP Ni NCs^a^	10.7	3.14	(29)
	Cu–Ag/GP	4.05	0.07	(39)
	Cu–Ni/GP	6.08	0.11	(39)
	Cu@Ni-NWs/G (5:1)	1.16	0.45	(40)
	Cu_3_Ni_2_	9.6	0.15	(41)
	Ni	1.3	2.83	this work
	Ni_7_Cu	8.3	18.04	this work
	Ni_3.5_Cu	7.0	15.21	this work
	Ni_1.75_Cu	21.0	46.65	this work
	NiCu	10.0	21.70	this work
	Cu	13.0	28.26	this work
3-nitrophenol	Ag/γ-Al_2_O_3_ calcinated	1.8	19.35	(71)
	Ni/γ-Al_2_O_3_ calcinated	0.5	2.94	(71)
	CoMoO_4_	21.8	0.05	(78)
	Ni/C black	9.8	0.53	(79)
	FCC Ni NCs^a^	′7.9	2.32	(29)
	HCP Ni NCs^a^	12.0	3.53	(29)
	Ni	0.06	0.13	this work
	Ni_7_Cu	12.0	26.10	this work
	Ni_3.5_Cu	4.4	10.87	this work
	Ni_1.75_Cu	11.8	25.65	this work
	NiCu	10.0	21.74	this work
	Cu	10.3	15.87	this work
2-nitrophenol	Ag/γ-Al_2_O_3_ calcinated	3.7	39.78	(71)
	Ni/γ-Al_2_O_3_ calcinated	1.5	8.82	(71)
	CoMoO_4_	4.55	0.01	(78)
	Ni/C black	9.9	0.53	(79)
	FCC Ni NCs^a^	4.5	1.32	(29)
	HCP Ni NCs^a^	25.6	7.53	(29)
	Cu–Ag/GP	1.21	0.02	(39)
	Cu–Ni/GP	1.11	0.02	(39)
	Ni	0.43	0.93	this work
	Ni_7_Cu	15.7	34.1	this work
	Ni_3.5_Cu	6.5	14.13	this work
	Ni_1.75_Cu	8.3	18.04	this work
	NiCu	14.0	30.43	this work
	Cu	6.8	14.78	this work

a Reaction at 50 °C.

Activity parameter *K* is commonly estimated to compare the catalytic activity of several samples by normalizing *k*_app_ to the amount of catalyst dispersed in the reactors for each experiment. The analysis of *K* values (Table 1) revealed that all bimetallic Ni_*x*_Cu_*y*_ nanostructures demonstrated relatively high catalytic activity in the reduction of NPS isomers, with the most effective catalyst being Ni_7_Cu, especially for the reduction of 2-NP and 3-NP molecules. Meanwhile, the Ni_1.75_Cu catalyst revealed the highest catalytic activity in the reduction of the 4-NP isomer. It is well-known that the reaction proceeds via the adsorption of the reagent molecule on the catalyst surface promoted by the electron-withdrawing of the nitro group in the NPS molecule. However, the position of the nitro group with respect to the O^–^ in the NPS isomer should affect the adsorption via electronic effects due to the inter- and intramolecular interactions. Moreover, the metallic species on the catalyst surface are the critical factor in hydrogen activation.^43,44^ Thus, the catalytic activity for the studied catalysts was firmly ruled by the capacity of the catalyst to stabilize the reagents and the presence of metallic atoms on the catalyst surface.

It may be proposed that the presence of oxidized Cu species is a key factor in the reduction of NPS isomers. As mentioned above, the catalysts were composed of nanospecies with a polycrystalline structure. In the Ni_7_Cu catalyst, the contribution of the Cu_2_O species was relatively high. Thus, the fast reduction of 2-NP on the Ni_7_Cu catalyst may be affected by the presence of oxidized species, which mitigates the intramolecular interactions in the isomer molecule, allowing its absorbance on the catalyst surface. In contrast, an intermolecular interaction is characteristic of the 4-NP isomer. In this case, metallic Cu or CuO species are required on the surface of the catalysts. Indeed, the Ni_1.75_Cu catalyst exhibited a huge amount of CuO species that was demonstrated by UV–vis and Raman spectroscopy results. It is well-known that CuO may be more quickly reduced into metallic Cu species than Cu_2_O.^77^ Thus, the reduction of 4-NP was ruled by the catalysts with a high contribution of CuO species, which rapidly transformed into metallic Cu species. Indeed, the order of the catalyst activity found for the 4-NP reduction was Ni_1.75_Cu > Cu > NiCu > Ni_7_Cu > Ni_3.5_Cu > Ni, which correlates well with the contribution of CuO species in the samples. Note that the estimated *K* value revealed comparable activity for all Ni_*x*_Cu_*y*_ and Cu catalysts in 3-NP reduction when neither inter- nor intramolecular interactions play a critical role.

To confirm the effect of oxidized species on catalytic activity, a different reaction protocol was applied, using 4-NP as the probe. Basically, the catalysts were pre-reduced with NaBH_4_, a strong reducing agent and reagent in this reaction, before the injection of 4-NP into the reaction cell. First, the UV–vis spectra of the catalysts in contact with NaBH_4_ were *in situ* monitored. The recorded spectra are presented in Figure S5 in the Supporting Information. It was found that the absorbance of the spectrum decreased in each case until the formation of the surface plasmon resonance characteristic of metallic Cu centered at ∼580 nm (see the purple faded arrows in Figure S5). The time required for the oxidized species to transform into metallic ones was almost three times greater for bimetallic catalysts with respect to the monometallic Cu catalyst. However, the oxidized species were still present in the catalysts (pink faded arrows in Figure S5). After 20 min of pre-reduction of the catalysts, 4-NP was injected into the cell. The catalytic activity in terms of *k*_app_ for NiCu and Cu catalysts is summarized in Figure S6 in the Supporting Information. It was demonstrated that the pre-reduction of samples caused a decrease of up to 60% in the *k*_app_ value. It may be proposed that the formation of metallic species in the bimetallic Ni_*x*_Cu_*y*_ and Cu catalysts under reduction conditions decreases their catalytic activity due to their possible agglomeration. An increase in the catalytic activity was observed for Ni_7_Cu catalysts only. This result will be discussed later.

Table 1 summarizes the catalytic activity of studied samples and those with similar compositions reported in the literature, tested under similar conditions. The data analysis revealed that the bimetallic Ni_*x*_Cu_*y*_ catalysts were characterized by better activity in the transformation of the NPS isomers than the catalysts reported in the literature.^39,41^ These results may be attributed to the particular sheet-like morphology of the prepared samples, promoting a high dispersion of the catalysts’ active sites. In contrast, the prepared Ni sample demonstrated low catalytic activity in the reduction of NPS isomers, which may be explained by the FCC crystalline structure of the nanomaterials (Figure 3). It was reported that Ni nanocrystals with an HCP structure are more active in the reduction of NPS isomers than those with an FCC structure.^29^ According to the literature data, the nature of the supports may affect the formation of the nonmetallic Ni particles, allowing the strong interaction of the nanoparticles with the support. As shown in ref (79). Ni/C catalysts demonstrate the same *K* value, independently of the NPS isomer used as a reagent. In contrast, the deposition of Ni nanoparticles on γ-Al_2_O_3_ results in a different order of catalytic activity: 4-NP > 2-NP > 3-NP.^71^ A similar effect was obtained for the presently prepared Ni catalysts characterized by the high contribution of nonmetallic Ni species (see Table 1). Thus, the contribution of the nonmetallic Ni species favored the transformation of NPS isomers. Note that the highest catalytic activity in the 2-NP reduction was obtained for the Ni_7_Cu sample. These results are comparable with those obtained for Ag/γ-Al_2_O_3_ catalysts,^71^ known as the best catalyst for the reduction of NPS isomers.

**Figure 9:**
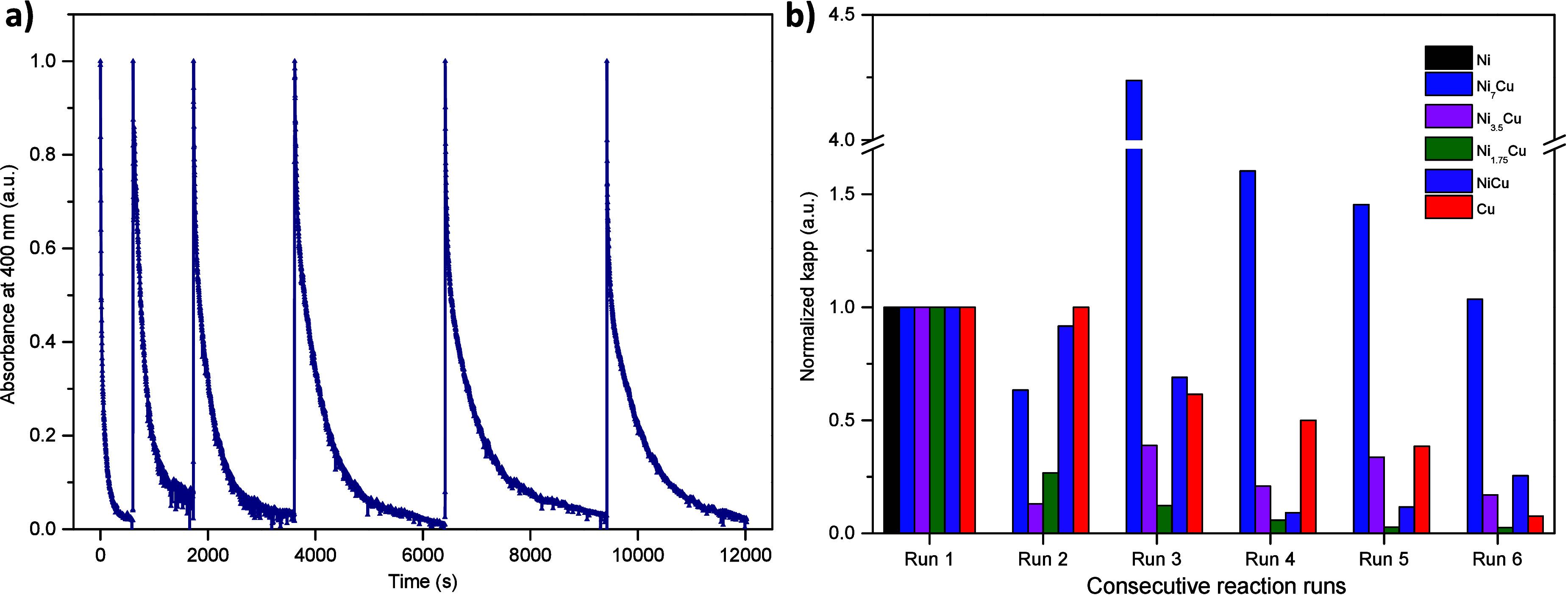
Catalyst stability in 4-NP reduction. (a) Absorbance changes at 400 nm for NiCu catalyst during consecutive catalytic runs. (b) Normalized *k*_app_ for Ni, Cu, and Ni_*x*_Cu_*y*_ catalysts during six consecutive catalytic runs.

Finally, the catalytic stability of the bimetallic Ni_*x*_Cu_*y*_ samples was studied in the reduction of 4-NP. For this purpose, the 4-NP dose was reinjected into the reactor immediately after its complete consumption in each catalytic run. Figure 9a shows the typical changes in the relative absorbance of the band centered at 400 nm. Complete consumption of 4-NP between each consecutive catalytic run was observed. Figure 9b presents the *k*_app_ values estimated for each consecutive catalytic run. It was found that the catalytic stability of the samples increased with the amount of Cu in the samples. However, partial deactivation of the catalysts after each consecutive run was observed and related to the Cu content in the samples. These results may be associated with the gradual transformation of the Cu^+^ species in contact with NaBH_4_. Note that the stability of pre-reduced samples was higher than that of nonreduced catalysts (see Figure S6 in the Supporting Information). Additionally, it is reported that Cu species may be released from the catalyst surface into the reaction medium.^80^ Hence, the deactivation of catalysts containing Cu was expected. In the case of the Ni catalyst, the intrinsic magnetism of the sample provoked its faster deactivation due to the accumulation of the Ni nanoparticles on the magnet, as described above.

A sudden increase in the catalytic activity in the reduction of 4-NP was observed for the Ni_7_Cu catalyst after the third catalytic run (Figure 9b). Additional experiments were carried out to explain this phenomenon. Prior to the catalytic test, the Ni_7_Cu catalyst was pre-reduced in the reaction medium by NaBH_4_ (a detailed kinetic analysis is presented in Figure S7). Indeed, the obtained results demonstrated that the pre-reduced Ni_7_Cu catalyst exhibited higher catalytic activity in the reduction of 4-NP compared to the as-prepared Ni_7_Cu catalyst. This difference may be explained by the presence of initially oxidized Ni and Cu species in the sample and their subsequent reduction in the reaction medium. Therefore, the increase in the catalytic activity of Ni_7_Cu catalysts after the third catalytic run was attributed to the abundance of oxidized Ni and Cu species, which was also confirmed by UV–vis and Raman spectroscopy results.

## 4 Conclusion

In the present work, nanosheets of Ni_*x*_Cu_*y*_ with different metallic molar ratios are easily obtained using a colloidal method. For the first time, the formation of a Ni_*x*_Cu*_y_* sheet-like morphology under relatively soft conditions is presented. Additionally, the used molar ratios of Ni:Cu reveal the effect of the decoration on the catalytic reduction of NPS, even in the presence of such a small amount of Cu. Prepared samples demonstrate the highest catalytic activity in the reduction of nitrophenol isomers compared to similar catalysts previously reported in the literature. The sheet-like Ni_*x*_Cu_*y*_ morphology enhances the catalytic activity via dispersion of the active sites. The presence of Ni–Cu alloys and CuO species on the catalyst surface allows the fast reduction of 4-NP. Meanwhile, the existence of Cu_2_O crystals minimizes the intramolecular interaction, promoting the adsorption of 2-NP and its subsequent transformation into 2-AP. The catalytic evaluation of the Ni_*x*_Cu_*y*_ samples reveals that the Ni_1.75_Cu catalyst is the most effective in the reduction of 4-NP. At the same time, the catalytic activity of the Ni_7_Cu catalyst in the reduction of 2-NP and 3-NP is comparable with that of noble-metal-based catalysts. Finally, it is demonstrated that pre-reduction of the catalysts with NaBH_4_, prior to the injection of 4-NP, results in lower catalytic activity in terms of *k*_app_ but high stability compared with the nonreduced samples. Therefore, the benefits such as low-cost production, high catalytic stability, and availability make the usage of Ni_*x*_Cu_*y*_ catalysts attractive for industrial applications in comparison with noble-metal-based catalysts.

## Supporting Information Available

TEM micrographs and EDX analyses of Cu nanoparticles and Ni_1.75_Cu sample, XPS analyses of Ni_7_Cu and Ni_*x*_Cu_*y*_ catalysts, crystallite sizes of catalysts, UV–vis spectra of the catalysts collected during their reduction in the presence of NaBH_4_, estimated *k*_app_ values for the reduction of 4-NP in the presence of pre-reduced catalysts during four consecutive catalytic runs, and kinetic analysis of Ni_7_Cu catalyst in the reduction of 4-NP (PDF)
