# Supplementary material for: Remarkable Enhancement of Catalytic Reduction of Nitrophenol Isomers by Decoration of Ni Nanosheets with Cu Species
Source: ACS Omega. 2024 Aug 27;9(36):37981–94. doi: 10.1021/acsomega.4c04762 (PMC11391462; doi:10.1021/acsomega.4c04762)
Supplement: Supplementary file 1 — ao4c04762_si_001.pdf [file ao4c04762_si_001.pdf]

# Remarkable Enhancement of Catalytic Reduction of Nitrophenol Isomers by Decoration of Ni Nanosheets with Cu Species

*Victoria Avalos-Ballester*[a], *Brenda Acosta*[a, b, *] *and Elena Smolentseva*[c, *]

[a] Coordinación para la Innovación y la Aplicación de la Ciencia y la Tecnología, Universidad Autónoma de San Luis Potosí, Álvaro Obregón 64, San Luis Potosí, S.L.P. 78000, México.

[b] Investigadora por México CONAHCYT, Coordinación para la Innovación y la Aplicación de la Ciencia y la Tecnología, Universidad Autónoma de San Luis Potosí, Álvaro Obregón 64, San Luis Potosí, S.L.P. 78000, México. *E-mail: brenda.acosta@uaslp.mx

[c] Universidad Nacional Autónoma de México Centro de Nanociencias y Nanotecnología, Km. 107 Carretera Tijuana a Ensenada, C.P. 22860, Ensenada, Baja California, México. E-mail: elena@ens.cnyn.unam.mx

## Supplementary Information

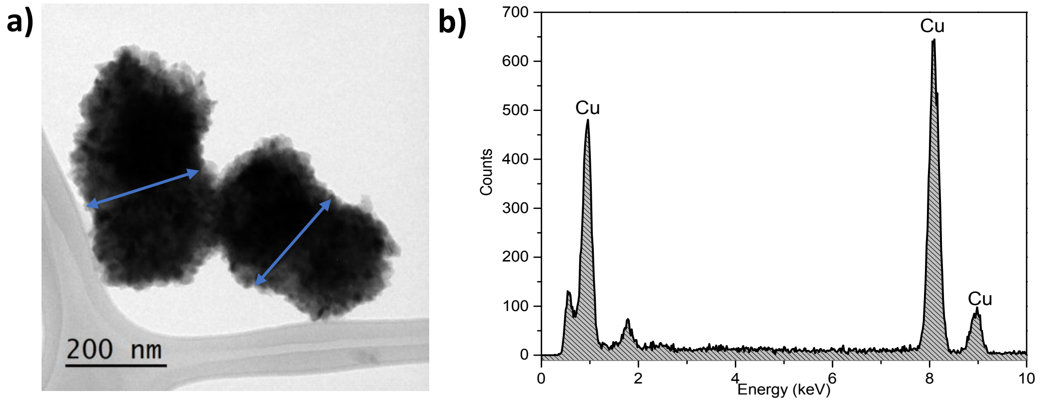


**Figure S1.** a) TEM Micrograph of Cu nanoparticles. b) EDX analysis of Cu nanoparticles.

Figure S1 shows a typical TEM image obtained for Cu nanoparticles. The formation of approximately 200 nm agglomerates was observed (measured in the direction of the blue line in Figure S1). These agglomerates were formed by smaller quasispherical nanoparticles around 10 nm in diameter. The morphology of formed Cu nanoparticles is typical for those Cu nanostructures synthesized with stabilizing agents.[1]

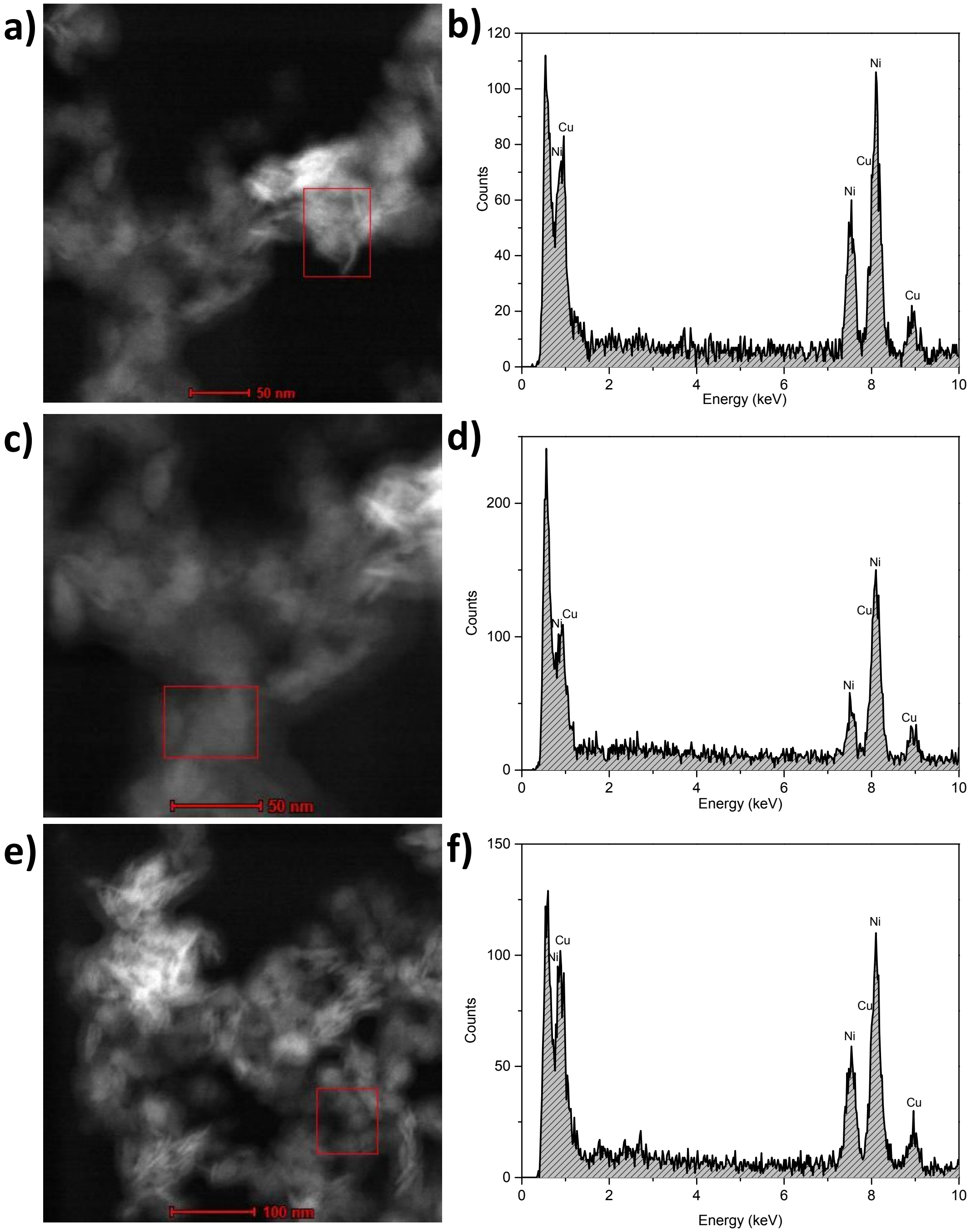


**Figure S2.** EDX analysis of three different zones for the $Ni_{1.75}Cu$ sample. a), c) and e) TEM micrographs. b), d) and f) Corresponding EDX spectrum collected from the zone marked with red box in each micrograph on the left.

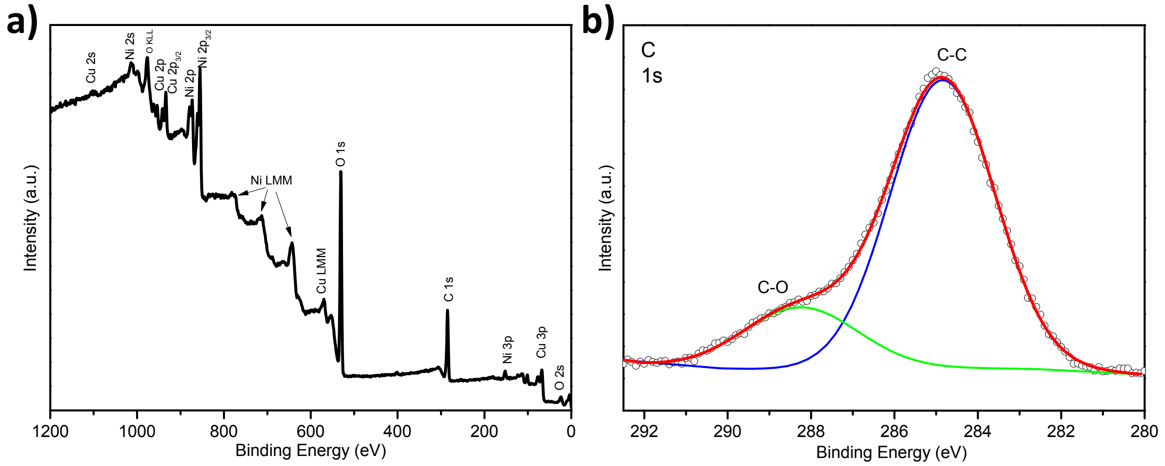


**Figure S3**. XPS analysis for the $Ni_7Cu$ catalyst. a) Survey and b) high-resolution spectra of C1s.

Figure S3 presents the Survey spectrum of the $Ni_{1.75}Cu$ sample obtained by the XPS analysis. The survey confirms the presence of Ni and Cu in the catalyst.

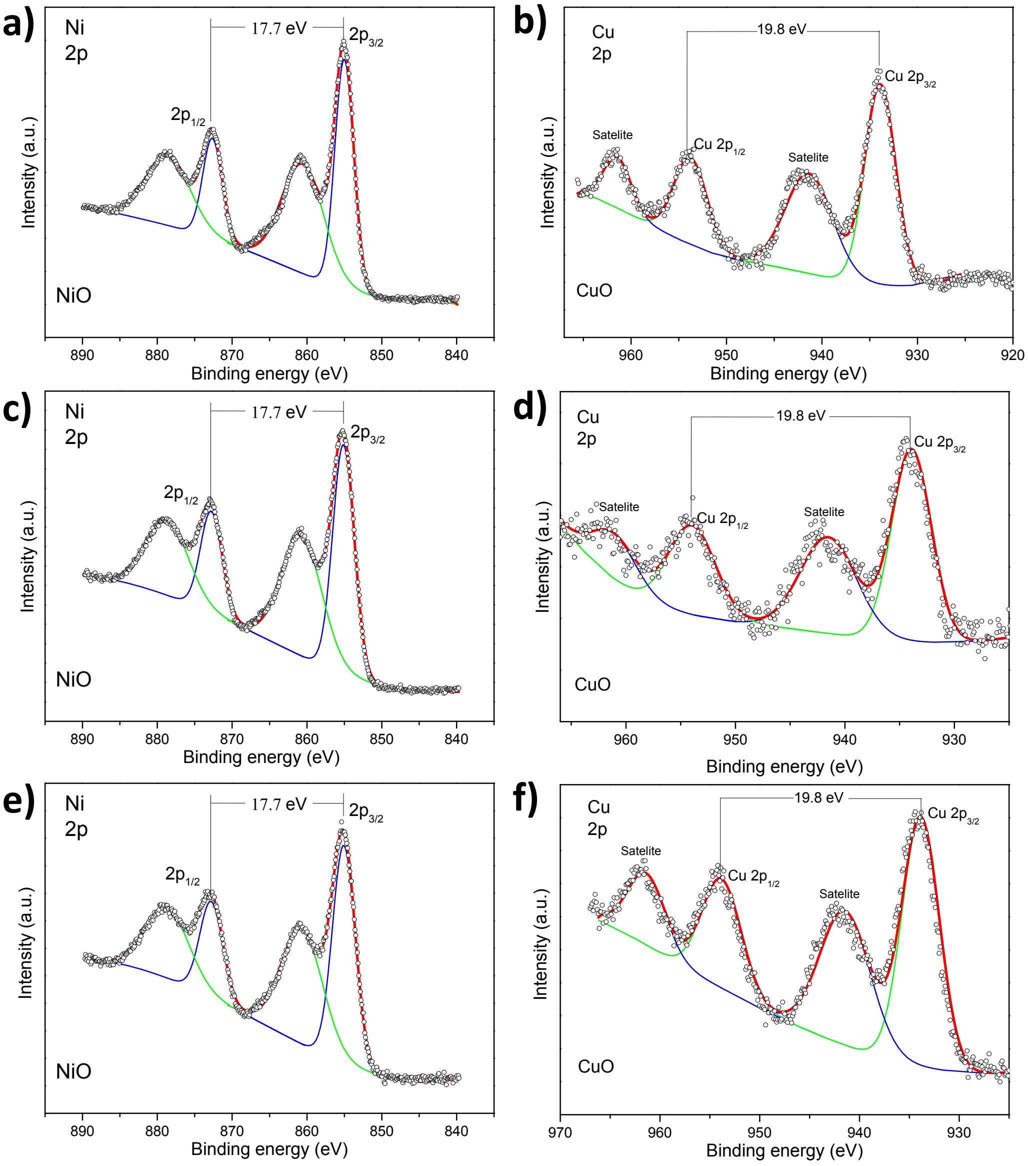


**Figure S4.** XPS analysis for $Ni_xCu_y$ catalysts. a), c) and e) Ni 2p and b), d) and f) Cu 2p high-resolution spectra for $Ni_7Cu$, $Ni_{3.5}Cu$ and NiCu samples, respectively. The red lines denote the fitting line for the experimental XPS spectra (circles), while the green and blue lines represent the deconvoluted spectra for the respective elements and satellites.

**Table S1.** Estimated crystallite size for the Ni, Cu and $Ni_xCu_y$ catalysts.

| Catalyst | Crystallite size (nm) |
|---|---|
| **Ni** | 18.74 |
| **$Ni_7Cu$** | 11.39 |
| **$Ni_{3.5}Cu$** | 16.37 |
| **$Ni_{1.75}Cu$** | 7.64 |
| **NiCu** | 15.67 |
| **Cu*** | 19.93 |

*(111) of metallic Cu FCC phase.

The crystallite size was estimated using the Scherrer equation[2] by taking the peak of Ni (111) crystallographic plane in each case.

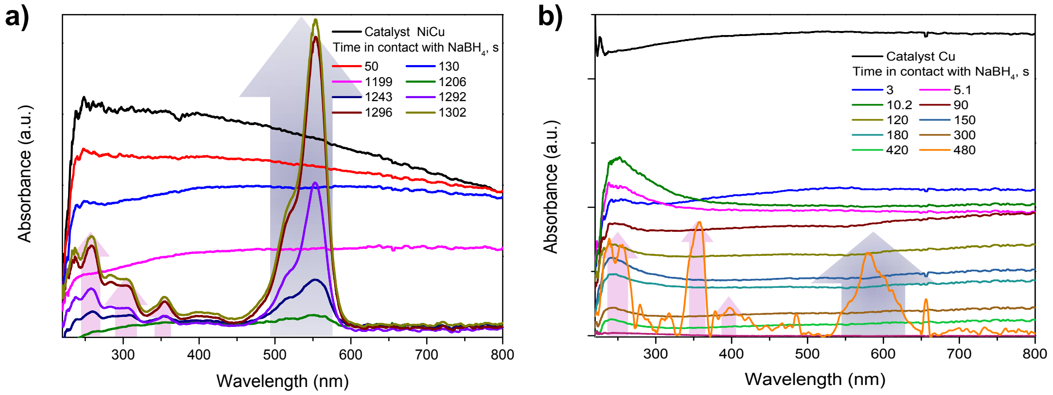


**Figure S5.** UV-vis spectra recorded *in situ* for a) NiCu and b) Cu catalysts in contact with $NaBH_4$. The pink faded arrows show the position of oxidized species meanwhile the purple faded arrows present the surface plasmon resonance of Cu.

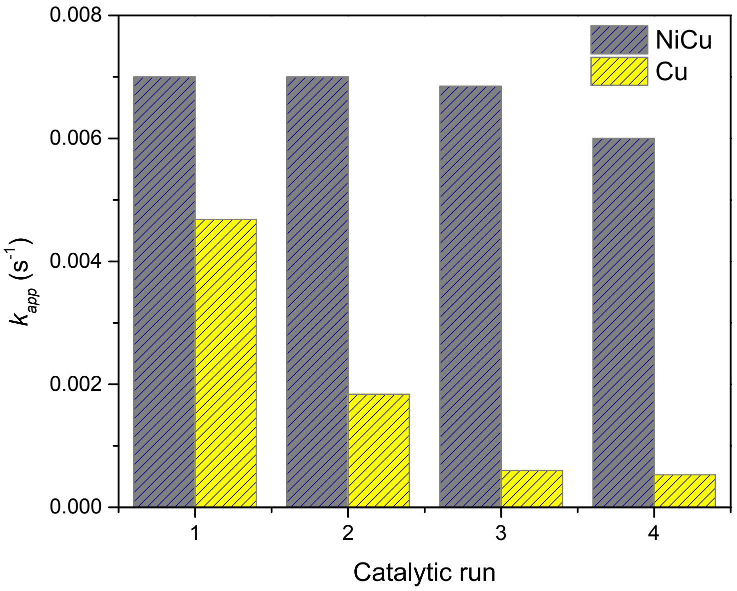


**Figure S6.** Estimated $k_{app}$ values for the reduction of 4-NP in the presence of the pre-reduced catalysts during four consecutive catalytic runs.

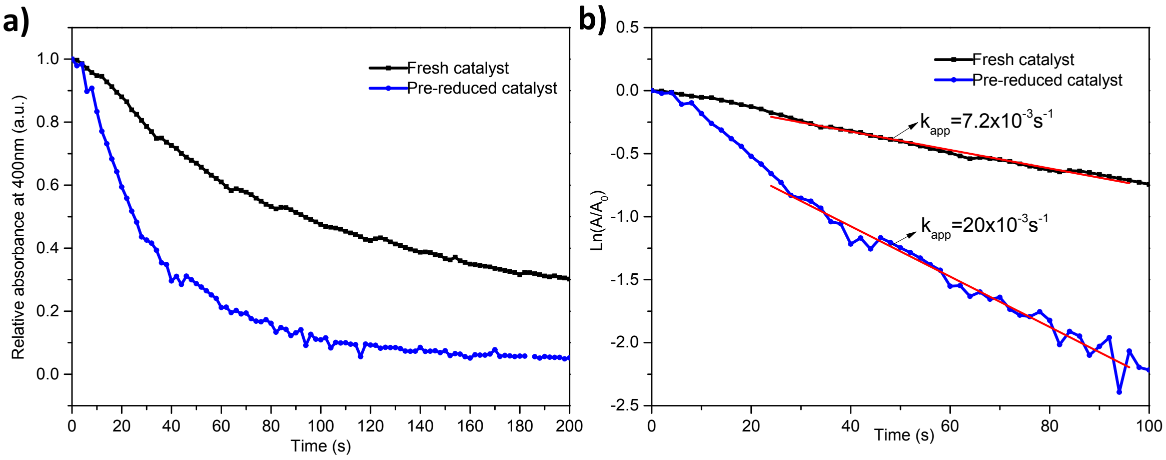


**Figure S7.** Kinetic analysis of $Ni_7Cu$ catalyst in the reduction of 4-NP. a) The changes in the relative absorbance at 400 nm. b) The plot of $\ln(A/A_0)$ vs reaction time.

# REFERENCES

(1) Sierra-Ávila, R.; Pérez-Alvarez, M.; Cadenas-Pliego, G.; Ávila-Orta, C. A.; Betancourt-Galindo, R.; Jiménez-Regalado, E.; Jiménez-Barrera, R. M.; Martínez-Colunga, J. G. Synthesis of Copper Nanoparticles Coated with Nitrogen Ligands. *J. Nanomater.* **2014**, *2014*. https://doi.org/10.1155/2014/361791.

(2) Scherrer, P. Nachrichten von Der Gesellschaft Der Wissenschaften Zu Göttingen. Mathematisch-Physikalische Klasse 1918, 2, 98–100.
